# Adaptive Traits Are Maintained on Steep Selective Gradients despite Gene Flow and Hybridization in the Intertidal Zone

**DOI:** 10.1371/journal.pone.0019402

**Published:** 2011-06-14

**Authors:** Gerardo I. Zardi, Katy R. Nicastro, Fernando Canovas, Joana Ferreira Costa, Ester A. Serrão, Gareth A. Pearson

**Affiliations:** CCMAR-CIMAR Laboratório Associado, Universidade do Algarve, Gambelas, Faro, Portugal; The University of Queensland, St. Lucia, Australia

## Abstract

Gene flow among hybridizing species with incomplete reproductive barriers blurs species boundaries, while selection under heterogeneous local ecological conditions or along strong gradients may counteract this tendency. Congeneric, externally-fertilizing fucoid brown algae occur as distinct morphotypes along intertidal exposure gradients despite gene flow. Combining analyses of genetic and phenotypic traits, we investigate the potential for physiological resilience to emersion stressors to act as an isolating mechanism in the face of gene flow. Along vertical exposure gradients in the intertidal zone of Northern Portugal and Northwest France, the mid-low shore species *Fucus vesiculosus*, the upper shore species *Fucus spiralis*, and an intermediate distinctive morphotype of *F. spiralis* var. *platycarpus* were morphologically characterized. Two diagnostic microsatellite loci recovered 3 genetic clusters consistent with prior morphological assignment. Phylogenetic analysis based on single nucleotide polymorphisms in 14 protein coding regions unambiguously resolved 3 clades; sympatric *F. vesiculosus*, *F. spiralis*, and the allopatric (in southern Iberia) population of *F. spiralis* var. *platycarpus*. In contrast, the sympatric *F. spiralis* var. *platycarpus* (from Northern Portugal) was distributed across the 3 clades, strongly suggesting hybridization/introgression with both other entities. Common garden experiments showed that physiological resilience following exposure to desiccation/heat stress differed significantly between the 3 sympatric genetic taxa; consistent with their respective vertical distribution on steep environmental clines in exposure time. Phylogenetic analyses indicate that *F. spiralis* var. *platycarpus* is a distinct entity in allopatry, but that extensive gene flow occurs with both higher and lower shore species in sympatry. Experimental results suggest that strong selection on physiological traits across steep intertidal exposure gradients acts to maintain the 3 distinct genetic and morphological taxa within their preferred vertical distribution ranges. On the strength of distributional, genetic, physiological and morphological differences, we propose elevation of *F. spiralis* var. *platycarpus* from variety to species level, as *F. guiryi*.

## Introduction

Speciation requires the evolution of reproductive isolation between interbreeding populations, thus preventing the homogenizing effects of gene flow. In addition, following secondary contact, hybridization can blur previously clear species boundaries. Models of speciation based on divergence between geographically isolated populations (i.e. allopatric speciation) have historically dominated the literature [1]. More recently however, a resurgence of interest in sympatric divergence has led to the development of many theoretical models for sympatric speciation, driven by disruptive selection while populations are still exchanging genes [for reviews see 2], [3], [4], [5], [6], [7].

Gene flow generally retards speciation by reducing genetic divergence among populations [8], [9], [10], although intermediate levels of gene flow may increase genetic variation and therefore adaptive divergence [11], [12], [13]. Despite having received less attention, small-scale spatial variation may lead to segregation of specific genotypes if natural selection is sufficiently intense to overcome the homogenizing effects of gene flow [e.g. 14], [15]. Under such conditions, short-scale gene flow is partly restricted between populations specifically adapted to microhabitats and maladapted to contrasting environments, as admixed individuals may experience reduced fitness and consequent elimination by selection [12].

The intertidal zone, where vertical gradients in emersion time produce strong variation in abiotic (e.g. desiccation, temperature extremes) and biotic (e.g. competition, predation pressure) conditions over very small (meters) spatial scales [16], provides an ideal environment for exploring questions concerning adaptive divergence and maintenance of ecotypes/species in the face of hybridization and gene flow. A classical example of habitat-driven divergence with gene flow on intertidal rocky shores is the case of the high/low shore ecotypes of the gastropod *Littorina saxatilis* [17], [18], that can mate and yield fertile intermediates [19], a possible example of parallel speciation [20], . Although in marine systems population connectivity and gene flow are potentially high, an increasing number of studies report greater population genetic structure in marine species at a smaller scale level than expected from life history alone [e.g. 22], [23], [24], [25].

The genus *Fucus* comprises of intertidal brown algae, it is the most species-rich within the family Fucaceae, and appears to have undergone recent radiation that has given rise to several closely related species [26], [27]. These brown algae are fascinating models for studies of local adaptation, ecological divergence and speciation for a number of reasons. On many intertidal shores, several coexisting species of algae overlap their vertical ranges according to their emersion abilities during low tide [for reviews see 28], [29]. For instance, *F. spiralis* (L.) individuals are exposed to air at low tide for longer than *F. vesiculosus* (L.) individuals. Laboratory studies have also shown small, but significant, differences in emersion tolerance between *F. spiralis* and *F. vesiculosus* [e.g. 30]. Variation in emersion times creates competition between early settlers of juveniles. For example, it has been shown that *F. vesiculosus* can extend its vertical range upshore when *F. spiralis* is removed [31], [32], while *F. spiralis* is competitively excluded by *F. vesiculosus* in the midshore region [33]. Moreover, these two taxa show contrasting mating systems, *F. spiralis* is hermaphroditic whereas *F. vesiculosus* is unisexual (i.e. dioecious taxon). In both species, planktonic larval phase is absent leading to rapid settlement very near parent algae [34].

Molecular phylogenies using traditional markers (nuclear ITS and mtDNA) have failed to discriminate between *F. vesiculosus* and *F. spiralis* [26], [27], although allelic frequencies of five microsatellites across a broad geographic area clearly demonstrate genetic isolation along currently accepted taxonomic lines [35]. Genetic isolation is very likely reinforced by mating system variation [36]; *F. vesiculosus* is dioecious (outcrossing), while *F. spiralis* is a hermaphrodite with high levels of inbreeding [36], [37]. Despite their different reproductive strategies and partial habitat segregation, hybridization occurs between *Fucus* taxa [34], [35], [36], [38], [39], suggesting that adaptive divergence under the different selective regimes encountered in the intertidal zone is stronger than the homogenizing effect of gene flow.

About a century ago, several *Fucus spiralis* morphotypes were described as morphological varieties, currently considered taxonomically equivalent [40], such as *F. spiralis* var. *platycarpus* (Thuret), first described over a century ago [41], and cited in more recent studies [42], [43]. Recently, genetic studies using microsatellite markers confirmed the existence of cryptic genetic divergence within *F. spiralis* [39]. These were described as *F. spiralis* Low and *F. spiralis* High because of their respective vertical distributions in the intertidal both occurring on average higher than *F. vesiculosus* [39]. Although the study of Billard et al. [39] was based on a random sampling design without morphological assignment of *F. spiralis* High and Low morphotypes, a subsequent assessment suggested that *F. spiralis* High and Low correspond morphologically to *F. spiralis* var. *typicus* [44, hereafter designated *F. spiralis* given its typical morphology] and *F. spiralis* var. *platycarpus* [41] respectively.

This study addresses the major question of persistence of genetic divergence in sympatry/parapatry despite gene flow, using as models two species and two varieties within one of those species, which occur along a sharp environmental gradient. We aimed to formally establish morphotype descriptions and the correspondence between morphology and genetic differentiation using previously identified diagnostic microsatellite markers [39]. We then used a common-garden design comparing trait means for emersion-stress resilience in order to test for differential selective constraint and habitat preferences along intertidal gradients. Finally, we used a multilocus phylogenetic analysis to resolve, for the first time, the relationships among sympatric *F. vesiculosus*, *F. spiralis*, and sympatric/allopatric *F. spiralis* var. *platycarpus*. Our data show significant differences in emersion stress resilience between morphotypes, despite extensive asymmetric introgression in *F. spiralis* var. *platycarpus* when in sympatry.

## Materials and Methods

**Figure 1:**
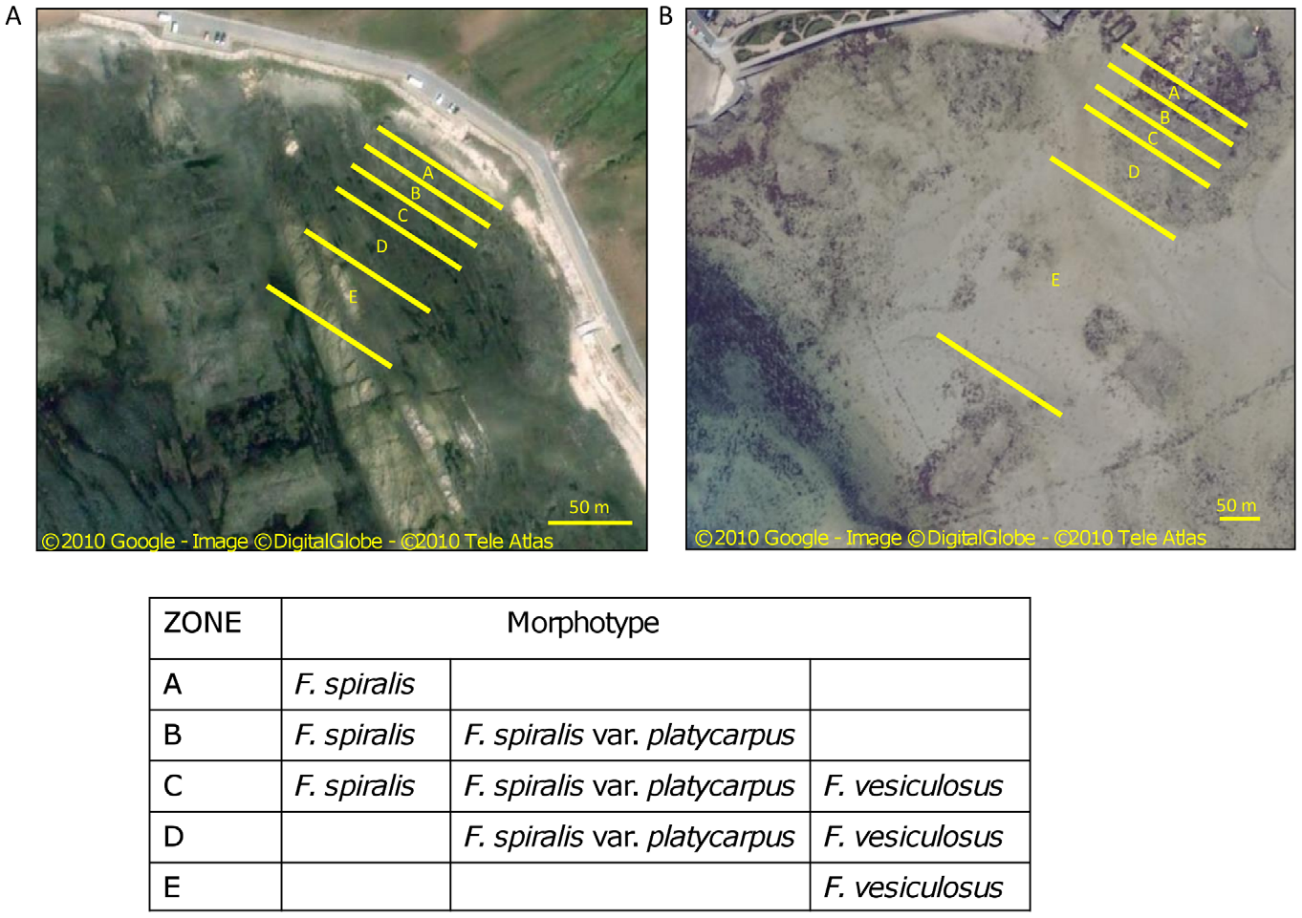
Zonation in *Fucus* zone. Division of *Fucus* zone into five areas based on distribution of the three morphotypes at (A) Viana, Portugal and (B) Roscoff, France.

From Northern Portugal northwards *F. vesiculosus* and *F. spiralis* occur in sympatry, with both species present on the open coast and in sheltered habitats (estuarine and coastal lagoons). From Northen Portugal southwards towards Morocco the two species have an allopatric distribution, with *F. vesiculosus* occurring only in estuarine and coastal lagoons and *F. spiralis* present only on the open coast. Sampling along the sympatric range (where the two taxa and *F. spiralis* var. *platycarpus* co-occur in the same habitat) was carried out in Viana do Castelo (hereafter shortened as Viana, 41°41′27″N, 8°50′57″W), Portugal, and in Roscoff (48°43′39″N 3°59′20″W), France, both in 2009. Based on previous morphological observations [39], [42] we sampled reproductive adult individuals of *F. vesiculosus*, *F. spiralis* and *F. spiralis* var. *platycarpus* co-occur [as defined in 39]. These three morphotypes showed a clear, partially overlapping, vertical zonation in the *Fucus* zone on both shores, which can be divided in five areas (Fig. 1): (A) the parapatric highest zone dominated by *F. spiralis*, (B) the zone inhabited by *F. spiralis* and *F. spiralis* var. *platycarpus* in sympatry, (C) a mixed area containing all three morphotypes, (D) the zone inhabited by *F. vesiculosus* and *F. spiralis* var. *platycarpus* in sympatry, (E) the lowest zone dominated by *F. vesiculosus* in parapatry.

In zone C at both locations, 50 individuals of each morphotype were sampled for genetic analysis using microsatellites, of which 10 were photographed for further characterization of morphological traits. At Viana only, out of the 50 individuals, 12 were brought to the laboratory to test for their physiological resilience to air exposure. In addition, further individuals of *F. spiralis*, *F. spiralis* var. *platycarpus*, and *F. vesiculosus* (12 each morphotype) were collected from all the other zones where each morphotype is present, and brought to the laboratory for similar physiological trials.

Of the 50 individuals of each morphotype collected at Viana, 5 individuals were characterized by sequencing cDNA fragments for 14 protein coding nuclear loci, together with 4 *F. spiralis* individuals from the allopatric, southern range (hereafter named *F. spiralis* var. *platycarpus* allopatric) collected in Vila Nova de Milfontes on the southwest coast of Portugal (37°43′08″N, 8°47′21″W).

Morphological traits of the southern morphotype were described using a population from Santa Eulália, southern Portugal (37°5′12″N, 8°12′59″W). At this sheltered location individuals reach a larger size that allows greater confidence in morphological characterization, relative to the typically stunted and wave-damaged individuals encountered on the more exposed southwest coast of Portugal. Therefore only undamaged individuals were collect to avoid morphological biased identification caused by wave brakeage.

### Exposure time

The median tidal height of each zone (A–E, see description above) was measured by recording the slope angle in the middle of each zone and the distance between each middle point and the lowest air exposed point during low tide (five measures each zone). Mean time of emersion during the year was calculated as the duration of emersion, according to the relative height on the shore, over 2008 in VC and RS using the SHOM database (http://www.shom.fr). Like others Fucoids, these species are perennial algae that do not undergo seasonal shifts in distribution [45].

### Morphology

**Figure 2:**
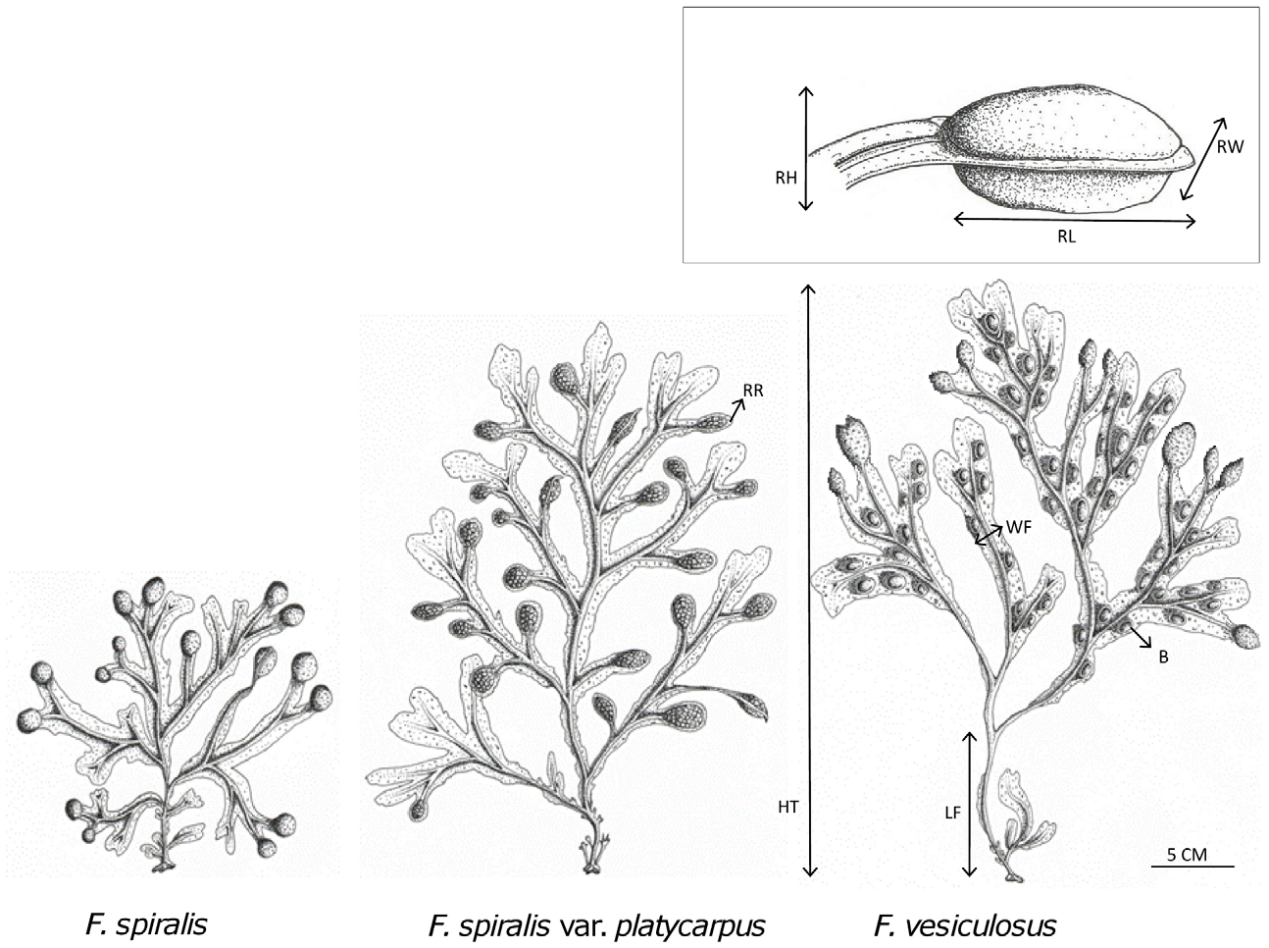
Drawings of morphotypes and illustration of morphological traits. Drawings of the three morphotypes. Added in the last drawing, illustration of measurements used to characterize morphotypes. Illustration of measurements used for the receptacles in the insert at the top. All traits are listed by abbreviation (see text for full explanation).

From the 50 individuals collected based on morphology, we haphazardly chose 10 individuals of each morphotype from Viana and from Roscoff. Digital photographs made in field were stored for morphometric analyses. Image analysis (ImageJ Processing and Analysis) was used to measure a suite of morphometric variables on each thallus (Fig. 2): thallus height (HT), length of the frond between the holdfast and the first dichotomy (LF); presence/absence of receptacle sterile rim (RR), air-bladders (B) and monopodial branching (M); width of apical frond (WF), receptacle height (RH), length (RL) and width (RW), ratio of receptacle width∶length (RW/L) and height∶length (RH/L). Five receptacles per thallus were selected haphazardly and measured for each variable and means per thallus were used as statistical replicates. We investigated the effect of morphotype (*F. vesiculosus*, *F. spiralis* var. *platycarpus*, *F. spiralis*) and site (Viana, Roscoff) using a MANOVA on the morphological variables. Post-hoc Tuckey tests were performed to assess the difference between the morphotypes (Statistica software version 8).

### Genetic analyses

#### Microsatellites

DNA was isolated from 5–10 mg of dried tissue with the CTAB method, but a silica filter plate (Milipore MultiScreen HTS, FB Cat. # MSFBN6B10) was used instead of the silica fines step. Microsatellite loci L20 and L78 [46], were used only as diagnostic markers between *F. spiralis*, *F. spiralis* var. *platycarpus* and *F. vesiculosus* in sympatry [see allele frequencies in 36], [39] with only the scope to test for correspondence with morphological classification. No attempt was made to use them for population genetic analysis. All PCR reactions were performed in a total volume of 15 µL containing 1× GOTaq polymerase buffer (Promega) with 2 mM of MgCl_2_, 0.03 mM of each dNTP, 0.17 µM of each Forward and 0.33 µM of each Reverse primer, 0.5 U GO *Taq* Polymerase and 5 µL of diluted DNA 1∶10. Amplifications were carried out on a Thermal Cycler 2720 (Applied Biosystems) using the following profile: initial denaturation at 94°C for 2 minutes; 35 cycles of 94°C for 20 seconds, followed by 35 seconds at 54°C for L20 and at 55°C for L78, 72°C for 40 seconds; and a final extension at 72°C for 20 minutes.

Structure software version 2.3 [47] analysis applies a Bayesian clustering approach and was used to identify the population structure inferred from microsatellites. We assumed a model in which there were from 1 to 6 populations (K clusters). Each K was replicated 20 times for 100000 iterations after a burn-in period of 50000, without any prior information on the population of origin of each sampled individual. The height of the modal value of ΔK distribution for the posterior probability of the data for a given *K* was used as an indicator of the strength of the signal detected by Structure and considered as the real number of K cluster [48]. Since inbreeding and selfing may induce linkage disequilibrium and Hardy-Weinberg disequilibrium which may not be suitable for assignment tests. Analyses were also performed with InStruct [49] which takes into account the possibility of selfing. We assumed a model where the number of cluster (*K*) was unknown and the population structure and selfing rates were inferred (lower and upper bound for *K* equal to 1 and 6, respectively). Clustering was running in 4 independent chains for the MCMC algorithm (100000 iterations after a burn-in period of 50000, without any prior information on the population of origin of each sampled individual).

#### Nuclear protein coding gene transcripts

From the 50 individuals collected in Viana, we haphazardly chose 5 individuals of each morphotype. An additional 4 individuals from the allopatric population of *F. spiralis* var. *platycarpus* were also included. RNA was isolated from fresh tissue of these individuals using the extraction method of Pearson et al. [50]. First strand cDNA was synthesized as follows; reaction mixtures containing 1 µg total RNA, 1 mM dNTPs and 5 µM oligo d(T) 18 were denatured at 70°C for 5 minutes. After ≥1 minute on ice, reverse transcriptase (RT) buffer (2× final concentration), MgCl_2_ (10 mM), DTT (0.1 M) ,RNase OUT and SuperScript™ III RT (Invitrogen) were added following the suppliers instructions, and the mix was incubated at 55°C for 1–2 hours, before inactivating the reaction at 80°C for 10 minutes.A total of 14 partial coding regions (see Supplementary Information S1) were selected for sequence analysis (BiP_HSP79_HS552: 186 bp; ClpB_ATPase: 507 bp; ClpC_HS598_HSP104: 600 bp; ClpP_protease: 399 bp; EIF3S6IP_HS700: 564 bp; HSP90_HS597: 309 bp; HSP90_HS870: 456 bp; Mpv_PMP22_1E12: 210 bp; Mpv_PMP22_HS544: 321 bp; STI1_6A15: 330 bp; STI1_HS0718: 459 bp; TCP1_delta: 183 bp; TCP1_epsilon: 354 bp; Mpv/PMP22_D025: 636 bp). The loci were chosen because they were found in ongoing studies to be phylogenetically informative within the genus *Fucus*, with conserved primer sequences that allowed relatively short amplification products and high-quality sequence reads. Specific primers were designed from Expressed Sequence Tag consensus sequences in *F. vesiculosus* [51; S1], using Primer3 software version 0.4.0 [52]. PCR was carried out in 20 µl reaction volumes containing 1–3 ul of first strand cDNA (1/40 dilution) as template, 1.5 mM MgCl_2_, 0.2 µM dNTPs, 0.5 µM of each primer and 1 U of *Taq* polymerase, with the following conditions: initial denaturation at 94°C for 3 minutes; 35 cycles of denaturation at 94°C for 20 seconds, annealing at 58°C for 90 seconds and a final extension at 65°C for 5 minutes. Products were sequenced on an ABI 3700 genetic analyzer. The resulting chromatograms were analyzed using CodonCode Aligner version 1.6.3 (CodonCode Corp., Dedham, Massachusetts, USA).

Sequences were aligned first by MAFFT version 6 using an iterative refinement method with global homology G-INS-i [53], then were corrected manually using Seaview software version 4 [54] and correspond to GenBank accessions (S1). Models of sequence evolution were selected by the Akaike Information Criterion as implemented in Modeltest version 3.7 [55] for each of the 14 partitions defined by each single gene: Hasegawa-Kishino-Yano model [HKY; 56] was found to be the most appropriate model for 1^st^, 6^th^, 7^th^, 10^th^, 12^th^ and 13^th^ partitions; Kimura 2-parameter [K2P; 57] for 2^nd^, 4^th^, 8^th^ and 9^th^ partitions; Tamura-Nei [TrN; 58] for 5^th^ and 11^th^; and General Time Reversible [GTR; 59] for 3^rd^ and 14^th^ partition. The combined data set was analyzed as one partition using the TrN model. Two phylogenetic reconstractions were performed, one with *F. vesiculosus*, *F. spiralis* and populations of *F. spiralis* var. *platycarpus* in sympatry and allopatry; the other excluding *F. spiralis* var. *platycarpus* in sympatry. *Fucus ceranoides* was used as outgroup species based on previous information about the phylogenetic relationships among species in *Fucus* genus [60].

Heterozygous sites were included and codified following standard nucleotide ambiguity codes. No gaps were detected in the sequences analyzed. To prevent wrong inference of haplotypes, *F*_ST_ values were calculated using polymorphism between sequences coded as SNPs information in Arlequin software v.3.5.1.2 [61].

Maximum likelihood bootstrap analysis with 9999 replicates was performed to infer the phylogenetic relationships for the combined data set using PhyML version 3.0.1 [62]. The substitution parameters were estimated over a neighbor-joining tree. Tree searching operations were set to best of nearest-neighbor interchange (NNI) and subtree pruning and regrafting (SPR).

Bayesian-based inference using the same alignment was performed with MrBayes version 3.1.2 [63]. For the partitioned analysis, the substitution model and branch length estimates were allowed to vary independently in each partition. General forms of these models were used since there is a specific recommendation against the use of fixed priors for a and I in the software manual in order to explore more efficiently different values of these parameters. The number of generations was set to 10^6^ with a sampling frequency of 100 generations in dual running process with four chains each run [64]. Majority rule consensus trees were computed in Phylip software version 3 [65] after discarding the first 25% of the trees (burnin), which were saved prior to MCMC convergence. Support for clades given by posterior probabilities was thus represented by the majority rule percentage.

### Physiological resilience to air exposure

Haphazard subsets of 12 individuals (each morphotype, Viana only) from the genotyped samples were acclimated in seawater (17°C) for seven days in 5 L tanks at low photosynthetic photon flux density (LL: PPFD of 30–50 µmol m^−2^ s^−1^) supplied by sodium vapour lamps. Additional samples of 12 individuals (each morphotype, Viana only) from the other zones where each morphotype is found were also collected and acclimated in the same way. Following acclimation, vegetative apical tips were cut and placed in 5 L tanks and kept for an additional 10 days. Half the seawater volume was replaced every 2 days throughout the acclimation period.

From each individual four tips were selected and duplicates were exposed in air at high photosynthetic photon flux density (PPFD: 250–300 ìmol m^−2^ s^−1^) at one of the following temperatures: (a) 33°C (±0.5°C), (b) 37°C (±0.5°C). An additional 40°C (±0.5°C) treatment was performed for individuals coming from zone C. Control treatments were kept in seawater at 17°C in LL.

Algal tissue was exposed to each treatment for 6 h and then allowed to recover under control conditions. After 1 h and 6 h recovery, photoinhibition of PSII maximum quantum yield (Fv/Fm) was measured with a chlorophyll fluorometer (FMS 2, Hansatech Instruments Ltd, UK). By relating the capacity for photochemical quenching (Fv) to the total fluorescence emission of closed PSII reaction centers (Fm), Fv/Fm is directly proportional to the quantum efficiency of PSII photochemistry [66], and its reduction from maximal values (0.7–0.8 in brown algae) is a sensitive and rapid screening tool for stress responses [67].

#### Analyses

The data were analysed using the PERMANOVA module [68], [69]. Unlike least-squares ANOVA, PERMANOVA requires no implicit assumptions about the underlying distribution (i.e. normality) or spread (i.e. variance) of the data within treatment groups and, whereas results are dependent upon the underlying distributions in the sense that observed differences between treatments may be because of differences between the means and/or spread, PERMANOVA does not assume either normality or homoscedasticity. For experiments (a) and (b), data were analysed under a nested design with treatment (control, treatment at 33°C and at 37°C), time of recovery (1 h, 6 h), morphotype (*F. spiralis*, *F. spiralis* var. *platycarpus*, *F. vesiculosus*) as fixed factors and zone (A, B and C for *F. spiralis*; B, C and D *F. spiralis* var. *platycarpus*; C, D and E for *F. vesiculosus*) nested in morphotype. Distance-based homogeneity of dispersion tests, tests of main effects and pair-wise tests on significant interactions were performed as recommended using 999 permutations. Therefore the number of times the permuted p-value was equal to or lying outside the 95% confidence interval was divided by the total number of permutations (999) and the resulted number taken as the permuted p-value.

## Results

### Exposure time

The biologically defined zones (based on morphotype presence) form a gradient of increasing exposure time from E to A in both Viana and Roscoff (Table 1). Average yearly estimated exposure times were between 12 and 18% lower at Viana compared with Roscoff, possibly producing greater emersion stress at Viana (i.e. higher maximum or average aerial temperatures).

**Table 1:** Emersion times.

Zone	Viana	Roscoff
A	63(±1)	81(±1)
B	55(±1)	72(±1)
C	49(±1)	67(±2)
D	34(±3)	50(±4)
E	29(±4)	41(±4)

Mean (±SD) estimated emersion times (%) for the 5 zones in Viana and Roscoff.

### Morphology

All *F. vesiculosus* individuals had bladders (B) while the other algae did not. The receptacle sterile rim (RR) and monopodial branching (M) were uniquely present in all *F. spiralis* var. *platycarpus* individuals. Because bladders, monopodial branching and receptacle sterile rim were diagnostic for a given morphotype, they had zero variance, and were thus excluded from the MANOVA (Table 2). Except for receptacle length (RL), which is similar in *F. vesiculosus* and in *F. spiralis* var. *platycarpus*, all other morphometric traits differed significantly between morphotypes. Results of this analysis revealed that effect of morphotypes was significant in all the remaining dependent variables (p<0.001) while the effect of site was not (p = 0.96). Results of post-hoc tests are shown in Table 2 for p<0.05. The allopatric *F. spiralis* var. *platycarpus* (Santa Eulália) shared the morphological traits of the sympatric *F. spiralis* var. *platycarpus*, namely the presence of a sterile rim around the receptacles, the relative proportions of the receptacles dimensions, and the monopodial branching (data not shown).

**Table 2:** Morphological traits.

	*F. spiralis*	*F. spiralis* var. *platycarpus*	*F. vesiculosus*	Post-hoc comparisons between morphotypes
M	absent	present	absent	…………………………………………
RR	absent	present	absent	………………………………………….
B	absent	absent	present	…………………………………………
LF	3.18(±0.58)	5.7(±0.62)	10.91(±1.51)	*F. vesiculosus*>*F. spiralis* var. *platycarpus*>*F. spiralis*
HT	25.29(±2.81)	34.11(±3.31)	43.48(±3.89)	*F. vesiculosus*>*F. spiralis* var. *platycarpus*>*F. spiralis*
WF	0.91(±0.053)	1.93(±0.09)	2.12(±0.07)	*F. vesiculosus*>*F. spiralis* var. *platycarpus*>*F. spiralis*
RH	0.9(±0.09)	0.73(±0.05)	0.4(±0.07)	*F. spiralis*>*F. spiralis* var. *platycarpus*>*F. vesiculosus*
RL	1.56(±0.1)	2.74(±0.16)	2.61(±0.17)	*F. spiralis* var. *platycarpus* = *F. vesiculosus*>*F. spiralis*
RW	1.1(±0.08)	1.53(±0.11)	1.27(±0.09)	*F. spiralis* var. *platycarpus*>*F. vesiculosus*>*F. spiralis*
RH/L	0.58(±0.06)	0.27(±0.02)	0.16(±0.03)	*F. spiralis*>*F. spiralis* var. *platycarpus*>*F. vesiculosus*
RW/L	0.72(±0.07)	0.57(±0.04)	0.48(±0.03)	*F. vesiculosus*>*F. spiralis* var. *platycarpus*>*F. spiralis*

Mean (±SD) morphological variables of 20 individuals each morphotype. Variables are listed by abbreviation (see text for full explanation). B, RR and M were recorded as presence and absence. Results of Tukey's post-hoc tests are included to determine differences between morphotypes.

### Genetic analyses

#### Microsatellites

**Figure 3:**
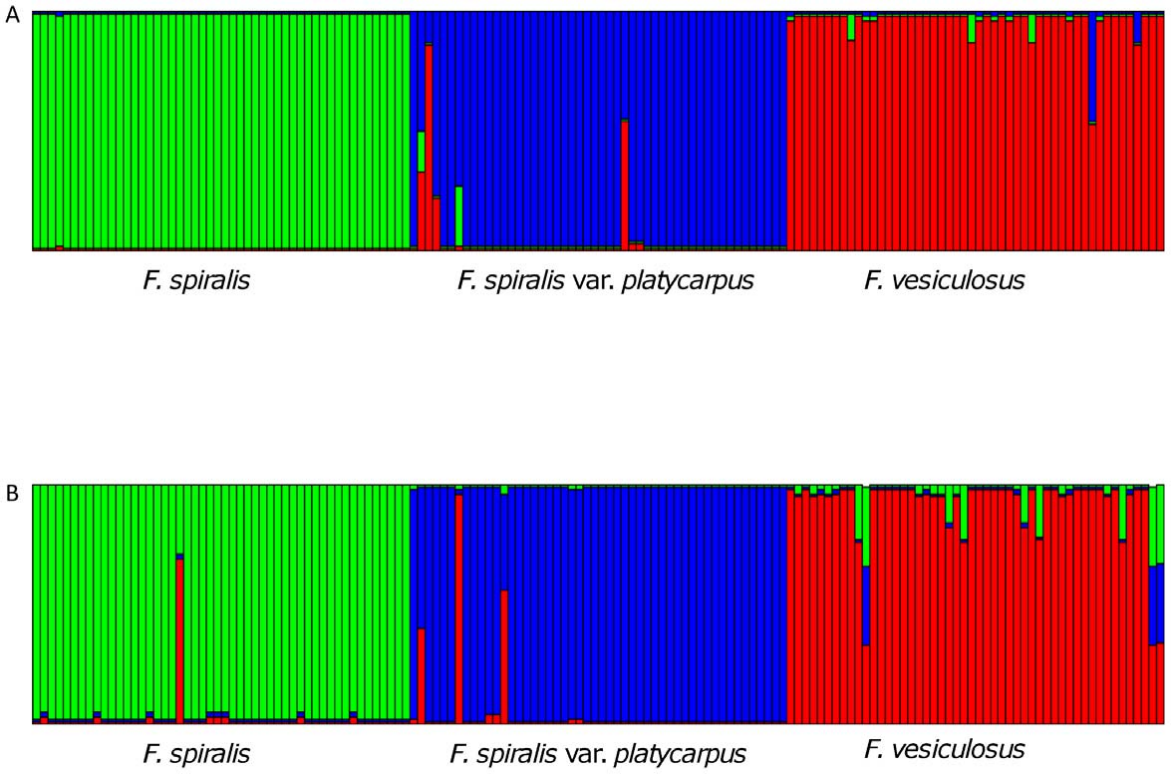
Results of the Bayesian analysis. Summary plot of the Bayesian analysis performed using STRUCTURE software on microsatellite data of samples from (A) Viana and (B) Roscoff, suggesting a K = 3 best describing the situation among samples. Each vertical bar represents a different individual and length color is proportional to the inferred cluster. All individuals belonging to a morphological entity are generally assigned to the same cluster.

Bayesian admixture analyses, implemented by both Structure software [47] and InStruct [49, results not shown], and based on two microsatellite loci with alleles previously identified as diagnostic for *F. spiralis* (*sensu lato*) and *F. vesiculosus* [36], produced K = 3 clearly defined groups corresponding to the *F. spiralis* and *F. spiralis* var. *platycarpus* morphotypes and *F. vesiculosus* at both locations. Nevertheless, several individuals morphologically identified as *F. spiralis* var. *platycarpus* and *F. vesiculosus* displayed genetic characteristics of another entity (Fig. 3a,b). Interestingly, most cases of morphological/genetic inconstancy appeared to involve *F. spiralis* var. *platycarpus* and *F. vesiculosus*, while only one clear case involved *F. spiralis* from Roscoff (Fig. 3b), and when the analysis was performed using InStruct, no cases of genetic inconstancy were observed in *F. spiralis* (data not shown).

#### Nuclear protein coding gene transcripts

Analysis of protein coding genes provided 29 variables sites, 15 of which were parsimony-informative. The highest divergence was found between *F. vesiculosus* and *F. spiralis sensu lato*, as shown by nucleotide differences and *F*_ST_ values that were significant between all *Fucus* taxa (Table 3).

**Table 3:** Inter-species average pairwise differences.

	*F. spiralis*	*F. spiralis* var. *platycarpus*	*F. vesiculosus*
*F. spiralis*	-	0.206/***	0.724/***
*F. spiralis* var. *platycarpus*	0.857/**	-	0.418/***
*F. vesiculosus*	0.665/***	0.714/*	-

*F*_ST_ values and their significance. *F. spiralis* var. *platycarpus* populations were considered in simpatry and allopatry (above diagonal) and only in allopatry (below diagonal). Significance codes: *P<0.001* “***”; *P*<0.01 “**”; *P*<0.05 “*”.

**Figure 4:**
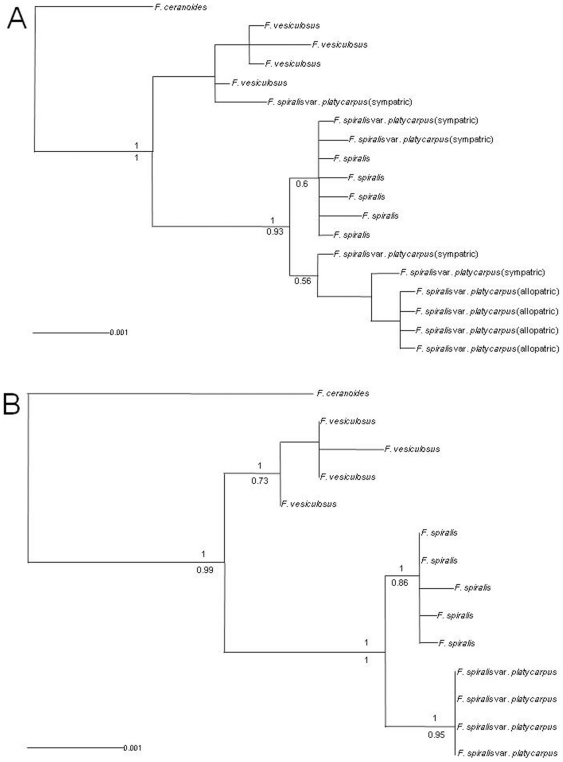
Phylogenetic tree. Phylogenetic relationships between *Fucus* morphotypes when *F. spiralis* var. *platycarpus* populations occur in sympatry and allopatry (a) or only in allopatry (b). Both the 50% majority rule percentage of support for clades given by Bayesian posterior probabilities from one million generation MCMC analysis (above) and the 50% majority rule consensus tree of maximum likelihood bootstrap (below) based on the 14 coding regions used. Both trees were rooted using *F. ceranoides* as outgroup species.

ML and Bayesian analyses yielded phylogenetic trees with the same topology (Fig. 4a,b), with slight differences in the branch support values from both algorithms. Consequently, in both approaches each of the three major clades contained individuals from the same entity and clearly differentiated *F. vesiculosus*, *F. spiralis* and allopatric *F. spiralis* var. *platycarpus*. In contrast, the sympatric *F. spiralis* var. *platycarpus* was polyphyletic, with two individuals grouping together with the allopatric *F. spiralis* var. *platycarpus*, two with *F. spiralis* and one with *F. vesiculosus* (Fig. 4a). This was confirmed in the tree excluding sympatric *F. spiralis* var. *platycarpus* where allopatric individuals were monophyletic (Fig. 4b).

### Physiological resilience to air exposure

**Figure 5:**
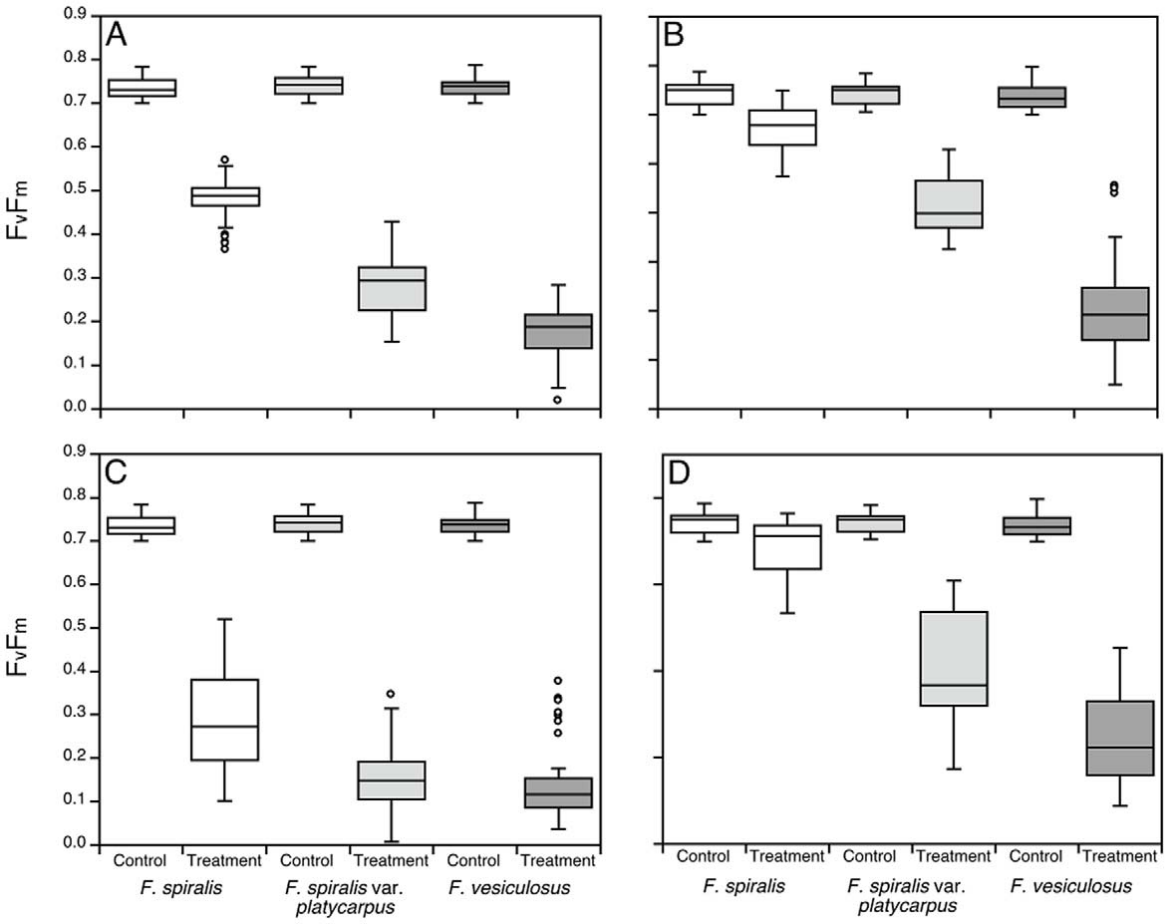
Physiological resilience to air exposure. Measurements of photoinhibition of PSII maximum quantum yield of *F. spiralis*, *F. spiralis* var. *platycarpus* and *F. vesiculosus* (zones pooled) after recovery from the following treatments: (A) and (B) morphotypes air exposed at 33°C (±0.5°C) at a PPFD of 250–300 µmol·m^−2^·s^−1^ (HL) after 1 and 6 hours of recovery respectively; (C) and (D) morphotypes air exposed at 37°C (±0.5°C) in HL after 1 and 6 hours of recovery respectively.

In experiments with taxa collected from different zones on the shore (3 zones each morphotype) temperature stress reduced Fv/Fm below that of controls, which did not differ between taxa. Zones did not have an effect for any of the taxa. However, while recovery occurred in all taxa after exposure at 33°C, with Fv/Fm increasing between 1 and 6 hours post-stress (Fig. 5a,b) significant differences in resilience between taxa were observed, where *F. vesiculosus*<*F. spiralis* var. *platycarpus*<*F. spiralis*. The results after 1 hour recovery from 33°C also suggest that either *F. spiralis* was less photoinhibited by this thermal stress, or recovered more rapidly. More severe stress at 37°C indicated that *F. vesiculosus* and *F. spiralis* var. *platycarpus* had a similar resilience after 1 hour recovery (p = 0.488), but after 6 h recovery *F. vesiculosus*<*F. spiralis* var. *platycarpus*. At both recovery times the resilience of *F. spiralis* was significantly greater than that of the other two morphotypes (treatment×time×species, p<0.001; Fig. 5c,d). Air temperatures of 37°C appear to be close to the tolerance limits of all 3 taxa, since at higher temperatures (40°C, only for morphotypes from zone C) little recovery of Fv/Fm occurred (data not shown).

The results show that resilience to emersion stress in the laboratory correlates with the observed distribution on the shore, with *F. spiralis*>*F. spiralis* var. *platycarpus*≥*F. vesiculosus*.

## Discussion

The genetic, ecological, and physiological factors that explain how gene flow persists against countervailing selection have seldom been studied [but see 8], [70], [71], [72]. Individuals locally adapted to one environment may be maladapted to contrasting environments and experience reduced fitness, leading to diminished rates of gene flow. When strong selection acts on a well-defined adaptive trait in the face of high gene flow, it offers an ideal opportunity to integrate population genetics and physiological genetics. It was shown recently that, rather than the classical *F. vesiculosus*/*F. spiralis* species pair, three genetically distinct fucoid morphology exist on some intertidal rocky shores of Portugal and France [39]. Their vertical distributions were distinct along intertidal gradients, and it was suggested that diversifying selection might play a role in maintaining genetic groupings despite evidence for gene flow between them [39]. This study investigated the role of emersion stress resilience as a potential selective agent. Morphometric analysis was used to assign sampled individuals to *F. vesiculosus*, *F. spiralis* var. *platycarpus* and *F. spiralis* while admixture analysis using microsatellite markers [as in 39] confirmed a robust correspondence between morphotype and genotype. We found robust physiological variation in emersion stress resilience that was consistent with the vertical distributions of the three genetic taxa in sympatry/parapatry on rocky shores. A phylogenetic analysis using SNPs from multiple genes, and including an allopatric population of *F. spiralis* var. *platycarpus* from southern Portugal, identified 3 monophyletic clades. However, sympatric/parapatric *F. spiralis* var. *platycarpus* was not monophyletic, suggesting that extensive asymmetrical introgression has occurred with local *F. vesiculosus* and *F. spiralis*. The phylogenetic analysis excluding sympatric *F. spiralis* var. *platycarpus* supported these findings, showing three well supported monophyletic clades.

Based on phylogenetic and phylogeographic evidence, the clade containing *F. vesiculosus* and *F. spiralis* has undergone recent divergence; previous phylogenies based on nuclear ITS [26] and mtDNA [27] markers were unable to discriminate among *F. spiralis* and *F. vesiculosus*, much less detect differentiation within *F. spiralis*. Nevertheless, microsatellite allele frequencies have demonstrated clear genetic differentiation and therefore a significant level of reproductive isolation between *F. spiralis* and *F. vesiculosus* across geographical sites [35]. The SNP loci we used for this study, recently developed from EST collections [73], unambiguously differentiate these two species. Moreover, the allopatric *F. spiralis* var. *platycarpus* from southern Iberia [which corresponds morphologically with *F. spiralis* Low as identified by Billard et al. [39]] appears to be a taxa phylogenetically distinct from *F. spiralis* and *F. vesiculosus*. In sympatry *F. spiralis* var. *platycarpus* morphotypes are polyphyletic, which is suggestive of extensive hybridization and introgression with both the other taxa. Although this conclusion remains tentative at present, the current data for the distribution of *F. spiralis* var. *platycarpus* support a model of secondary contact in the northern range. Spatially explicit model simulations [74] support the idea that introgression is asymmetric from local into the invading or expanding species, as our data suggest since *F. vesiculosus* and *F. spiralis* morphotypes are monophyletic in sympatry, and showed no evidence of introgression. This is supported by *F. vesiculosus* mitochondrial haplotypes having integrated into other *Fucus* (*F. ceranoides*, *F. spiralis*, *F. vesiculosus*) during postglacial re-colonization of Northern Europe [60], [75]. Several studies have shown that species barriers in *Fucus* remain permeable to gene flow, despite different reproductive strategies and partial habitat segregation [35], [36], [37], [38], [76], [77]. While it is generally thought that the homogenizing effect of gene flow counteracts the diversifying effect of local selection [78], gene flow under certain conditions may facilitate adaptive divergence via the spread of beneficial mutations among populations, by dampening stochastic variation that reduces fitness, or by increasing genetic variation (e.g. in inbred populations/species) that can enhance adaptive potential [reviewed in 12] and perhaps produce hybrid forms [79]. Here we show that selective regimes in the intertidal due to steep gradients in emersion time are reflected in physiological trait means for emersion resilience for the three *Fucus* genetic taxa, overcoming the homogenizing effects of gene flow.

Intertidal organisms are regularly covered and uncovered by the movement of tides that subject them to a transition from aquatic to terrestrial conditions. The upper distributional limits of many rocky intertidal organisms are thought to be set by some aspect of thermal and/or desiccation stress related to aerial exposure at low tide [30], [80], [81 reviewed by 28], . This is particularly true for sessile and sedentary organisms that are not capable of moving to evade environmental stresses imposed during low tide exposure to air (emersion). Several studies have shown that life in the high intertidal involves adaptation responses such as increased thermal resistance [82], heat stability of key metabolic enzymes [83], increased extracorporeal water storage, reduced evaporation [82], [84] and stress-induced expression of heat stress proteins [85]. We show that the three *Fucus* genetic taxa are partially segregated along the intertidal at both sampling sites and that their vertical distribution along the intertidal mirrors the gradient in the percentage of time out of water throughout a year. During low tide, temperature and desiccation interactively stress intertidal algae. An ecological trade-off occurs in which the protective effect of desiccation, that increases thermo-tolerance, comes at the cost of a decrease in photosynthesis [e.g. 86]. Hence algae at higher shore levels spend more time dry than similar species lower on the shore. The latter can potentially remain photosynthetically active for longer periods but are less adapted to high temperatures. Laboratory experiments showed that when comparing physiological performances of *Fucus* genetic taxa in response to air exposure at 33°C and 37°C, *F. spiralis* had the greatest resilience followed by *F. spiralis* var. *platycarpus* and then *F. vesiculosus*. Comparative physiological resilience to emersion between the three morphotypes mirrored their vertical distributions, confirming the crucial role of emersion-induced stress in setting upper vertical limits in the intertidal zone. No intraspecific differences in recovery rates of adult algae from different intertidal heights were detected after acclimation in common garden experiments, suggesting that (intraspecific) local adaptation is null or minor compared with interspecific variation [but see 24], [51].

Mating system variation can strongly restrict gene flow within and among conspecific populations and among closely related hybridizing species [e.g. 87], [88], [89], [90], leading to reproductive isolation and therefore possible speciation [91], [92]. *F. spiralis* (*sensu lato*) and *F. vesiculosus* show contrasting, species-diagnostic mating systems: *F. spiralis* individuals are hermaphroditic with a high degree of selfing [37], while *F. vesiculosus* is dioecious (obligately outcrossing). Thus, selfing in *F. spiralis*, as well as limited gamete dispersal capacities [90], [93], [94] and different release timing [95], most probably contribute to species integrity. Mating system alone however, may be inadequate to explain the asymmetric introgression of both *F. spiralis* and *F. vesiculosus* nuclear genomes into *F. spiralis* var. *platycarpus* (Fig. 4a) collected in the sympatric zone (C), since both *F. spiralis* genetic taxa share the same mating system. As mentioned above, asymmetric introgression from local to invading species may be common for neutral alleles during range expansion, as inferred from model simulations and literature surveys [73]. Further studies will be necessary to confirm the pattern observed with larger sample sizes, and to determine the possible contributions to introgression of, e.g. differential gamete release timing [e.g. 95], mating-system dependent variation in reproductive synchrony [96], or patterns of hybrid fitness and backcross ability [97], [98], [99].

The distinct morphotypes studied here could be unambiguously assigned to genetically distinct clusters with the two microsatellite loci used in this study. Together with the microsatellite data, morphological variation between entities showed similar pattern in North Portugal and in France indicating consistency across latitudinal scale. Other than receptacle length, which is similar in *F. vesiculosus* and in *F. spiralis* var. *platycarpus*, all other morphometric traits differ significantly between morphotypes. In particular, presence of bladders was typical of *F. vesiculosus* while occurrence of sterile margins in receptacles (rims) was a distinctive feature of *F. spiralis* var. *platycarpus*. These two morphological traits are visually immediate and do not require measurement, therefore they are ideal diagnostic features for field morphotyping. Populations of *F. vesiculosus* without bladders have been recorded on very exposed sites [e.g 100], however, in sites where bladders occur, like in most populations of *F. vesiculosus*, they are diagnostic for the species relative to other congenerics.

Morphological description and drawings in Perez-Ruzafa [42] of the two *F. spiralis* morphotypes are coherent with our findings. Many marine organisms have morphologies that reflect their physical environment and some of the best examples of morphological variation in marine organisms with respect to physical forces come from studies of benthic macroalgae [e.g. 101], [102], [103], [104], [105]. Although not known to occur in the *Fucus* genus, thallus morphology can also play a role in the algae's ability to withstand temperature and desiccation stress when exposed to air at low tide [106], [107]. Future studies may help to understand if the typical morphology of the three morphotypes could play a role in their ecological success along their shore height ranges.

We identify physiological, morphological, and genetic (at coding regions of multiple loci) differentiation between the genetic taxa *F. spiralis*, *F. spiralis* var. *platycarpus* and *F. vesiculosus*. We found that *F. spiralis* var *platycarpus* occurs in allopatry in southern Portugal, and in allopatry it was shown to form a monophyletic group distinct from *F. spiralis*. We therefore propose elevation of *F. spiralis* var. *platycarpus* [41] to the species level *F. guiryi*. In sympatry, *F. guiryi* showed strong signals of asymmetric introgression with both local species. Ongoing gene flow across permeable species boundaries has not been sufficient to disrupt adaptive physiological traits associated with emersion-stress resilience. We conclude that the steep vertical selective gradient spanning the intertidal zone is sufficiently strong to facilitate small-scale local adaptation and consequently maintain morphological and physiological species traits, even in the face of extensive (neutral) gene flow.

***Fucus guiryi*** Zardi, G.I., Nicastro, K.R., Serrão, E.S., Pearson, G.A. sp. nov.

*Diagnosis (Latin)*: Species nova *Fuci*. *Fuco vesiculoso* L. forma similis sed receptaculis hermaphroditis, ora sterili receptaculi, ramificatione monopodiali, absentia vesicarum; thallo breviore et longitudine frondis inter hapteron et primam dichotomiam breviore; fronde apicali angustiore; receptaculis altioribus latioribusque; proportione majore latitudinis receptaculorum cum longitudine et altitudinis receptaculorum cum longitudine; positione altiore inter accessum et recessum aestuum et tolerantia aeris majore; et transcriptis genum nuclearium ad proteina facienda (BiP1, clpB, clpC, clpP, eIF3l, HSP90_1, HSP90_2, PXMP2/4_2, mpv17l2, TTC1, STI1, CCT4, CCT-epsilon, PXMP2/4_1) et locis microsatellitum distinguenda (L20, L78). Fuco spirali L. quoque similis sed ora sterili receptaculi et ramificatione monopodiali; thallo longiore et longitudine frondis inter hapteron et primam dichotomiam longiore; fronde apicali latiore; receptaculis minus altis, longioribus, et latioribus; proportione minore latitudinis receptaculorum cum longitudine et altitudinis receptaculorum cum longitudine; positione inferiore inter accessum et recessum aestuum et tolerantia aeris minore; et transcriptis genum nuclearium ad proteina facienda (BiP1, clpB, clpC, clpP, eIF3l, HSP90_1, HSP90_2, PXMP2/4_2, mpv17l2, TTC1, STI1, CCT4, CCT-epsilon, PXMP2/4_1) et locis microsatellitorum distinguenda (L20, L78).

*Diagnosis (English)*: New species of the genus *Fucus*. Similar in morphology to *Fucus vesiculosus* L. but distinguished from it by hermaphroditic receptacles, the presence of a receptacle sterile rim, monopodial branching, and the absence of bladders; by shorter thallus and shorter frond length between the holdfast and the first dichotomy; by less wide apical frond; by higher and wider receptacles; by higher ratio of receptacle width∶length and receptacle height∶length; by higher intertidal zonation and physiological resilience to air exposure; by differences in the nuclear protein coding gene transcripts (BiP1, clpB, clpC, clpP, eIF3l, HSP90_1, HSP90_2, PXMP2/4_2, mpv17l2, TTC1, STI1, CCT4, CCT-epsilon, PXMP2/4_1) and microsatellite loci (L20, L78). Also similar in morphology to *Fucus spiralis* L. but distinguished from it by the presence of a receptacle sterile rim and monopodial branching; by longer thallus and frond between the holdfast and the first dichotomy; by wider apical frond; by shorter receptacle height; longer receptacle; wider receptacle; by smaller ratio of receptacle width∶length and receptacle height∶length; by lower intertidal zonation and physiological resilience to air exposure; by differences in the nuclear protein coding gene transcripts (BiP1, clpB, clpC, clpP, eIF3l, HSP90_1, HSP90_2, PXMP2/4_2, mpv17l2, TTC1, STI1, CCT4, CCT-epsilon, PXMP2/4_1) and microsatellite loci (L20, L78).

*Holotype:* Praia da Amoreira, Aljezur, SW Portugal; March 20th, 2011; Gareth Pearson GALW15586.

*Isotypes:* GALW15587, GALW15588, GALW15589, GALW15590, GALW15591.

*Etymology*: Named in honor of Michael Guiry, in recognition to his great contribution to phycology by creating AlgaeBase.

*Habitat*: Marine, intertidal. Where it co-occurs with *Fucus spiralis* and *Fucus vesiculosus* on the same shore, average distributional shore height typically in between these two species.

*Distribution*: Morocco, Canary Islands, Atlantic Spain, Portugal, France, north to Britain and Ireland and probably Scandinavia.

*Synonymy: Fucus platycarpus* Thuret, *Annales des Sciences Naturelles, Troisième série, Botanique*, 16: 9, pl. II (1851), *nom. illeg. non F. platycarpus* Turner *Fuci* vol. 3: 23, pl. 144 (1809–11) = *Botryoglossum platycarpum* (Turner) Kützing.

*Fucus spiralis* var. *platycarpus* Batters *Journal of Botany, British and Foreign* 40 *(Suppl.)*: 50, treated in Pérez-Ruzafa [42] as a new name.

*Valid publication*: The electronic version of this document in itself does not represent a published work according to the International Code of Botanical Nomenclature [ICBN; 108] and hence the new names contained in the electronic version are not effectively published under the provisions of the ICBN from the electronic version alone. Accordingly, a separate edition of this document has been produced by a method that assures numerous identical printed copies, and those copies were simultaneously distributed (on the publication date noted on the first page of this article) for the purpose of providing a public and permanent scientific record, in accordance with Art. 29 of the ICBN [108]. Copies of the print-only edition of this article were distributed on the publication date to botanical or generally accessible libraries of the following institutions (BM, C, DBN, GALW, PC, UC, US, LINN, B, NSW; http://sweetgum.nybg.org/ih/ for herbarium acronyms). A separate print-only edition of the article is available on request from PLoS (Public Library of Science) by sending a request to *PLoS ONE*, Public Library of Science, 1160 Battery Street, Suite 100, San Francisco, CA 94111, USA along with a check for $10 (to cover printing and postage) payable to “Public Library of Science”. No Unique Digital Identifier was available at the time of writing.

## Supporting Information

Supporting Information S1 **Nuclear protein coding gene transcript descriptions.** Annotations of coding region transcripts used in this study. P indicates partition number for each region used in mixed analyses. Total and used length expressed in base pairs (bp) and aminoacids (aa), as well as primer sequences and accession numbers, are shown.
(PDF)
