# Supplementary material for: Adaptive Traits Are Maintained on Steep Selective Gradients despite Gene Flow and Hybridization in the Intertidal Zone
Source: PLoS One. 2011 Jun 14;6(6):e19402. doi: 10.1371/journal.pone.0019402 (PMC3114782; doi:10.1371/journal.pone.0019402)
Supplement: Supporting Information S1 — Nuclear protein coding gene transcript descriptions. Annotations of coding region transcripts used in this study. P indicates partition number for each region used in mixed analyses. Total and used length expressed in base pairs (bp) and aminoacids (aa), as well as primer sequences and accession numbers, are shown. (PDF) [file pone.0019402.s001.pdf]

P	Gene name	Locus name	Description	length bp	length aa	used length	Primers (5'-3')	Accession numbers
1	BiP1	HS552	78 kDa glucose-regulated protein homologue	303	101	186	F CCTGTGTGCGTCTCGCCTCTTTTC<br>R GTCAAGGACCGCATCGAC	GU984820, GU984821, GU984822, GU984823, GU984824, GU984825, GU984826, GU984827, GU984828, GU984829, GU984830, GU984831, GU984832, GU984833, GU984834, GU984835, GU984836, GU984837, GU984838
2	clpB	-	casein lytic proteinase	669	223	507	F GCGTCAGTAGCTTCCAAAGT<br>R CTGCCTACATGTTCGACACC	GU984877, GU984878, GU984879, GU984880, GU984881, GU984882, GU984883, GU984884, GU984885, GU984886, GU984887, GU984888, GU984889, GU984890, GU984891, GU984892, GU984893, GU984894, GU984895
3	clpC	HS5978	ATP-dependent Clp protease ATP-binding subunit clpA	639	213	603	F CTCCACCGAAGCCAATTCCTTGTG<br>R CGTGCTCAAATGGGTGCTA	GU984896, GU984897, GU984898, GU984899, GU984900, GU984901, GU984902, GU984903, GU984904, GU984905, GU984906, GU984907, GU984908, GU984909, GU984910, GU984911, GU984912, GU984913, GU984914
4	clpP	-	ATP-dependent clp protease, proteolytic subunit clpP	462	154	399	F TGCGAACTGAGAAATGTGCGATGTC<br>R AAACAATGACCCGGAGAAAG	GU984915, GU984916, GU984917, GU984918, GU984919, GU984920, GU984921, GU984922, GU984923, GU984924, GU984925, GU984926, GU984927, GU984928, GU984929, GU984930, GU984931, GU984932, GU984933
5	eIF3l	HS700	eukaryotic translation initiation factor 3, subunit 6 interacting protein	573	191	564	F GTGTCCACGACGAGCCCATAAACG<br>R AAACGGCGGACAAGATGAT	GU984934, GU984935, GU984936, GU984937, GU984938, GU984939, GU984940, GU984941, GU984942, GU984943, GU984944, GU984945, GU984946, GU984947, GU984948, GU984949, GU984950, GU984951, GU984952
6	HSP90_1	HS597	Heat shock protein 90 family	303	101	309	F CTACGCGCCGTCTCGAAAACAGAC<br>R CTCAGGACAGCGGAGCAT	GU984953, GU984954, GU984955, GU984956, GU984957, GU984958, GU984959, GU984960, GU984961, GU984962, GU984963, GU984964, GU984965, GU984966, GU984967, GU984968, GU984969, GU984970, GU984971
7	HSP90_2	HS870	Heat shock protein 90 family	513	171	456	F CGACCACCTCTGCATCCTTCACC<br>R CAAGCCATCGGAGAGTACAAG	GU984972, GU984973, GU984974, GU984975, GU984976, GU984977, GU984978, GU984979, GU984980, GU984981, GU984982, GU984983, GU984984, GU984985, GU984986, GU984987, GU984988, GU984989, GU984990

8	PXMP2/4_2	D025	PXMP2/4 family protein 2	639	213	636	F TGCCCCCTTCACACATCCCAACT<br>R CCAACATGCCCAAGAAAATC	GU985029, GU985030, GU985031, GU985032, GU985033, GU985034, GU985035, GU985036, GU985037, GU985038, GU985039, GU985040, GU985041, GU985042, GU985043, GU985044, GU985045, GU985046, GU985047
9	mpv17l2	HS544	Mpv17-like protein	327	109	321	F AAACTACGGTTGCCGCACTCAACG<br>R CGATCAGACCATCTGGAACC	GU984991, GU984992, GU984993, GU984994, GU984995, GU984996, GU984997, GU984998, GU984999, GU985000, GU985001, GU985002, GU985003, GU985004, GU985005, GU985006, GU985007, GU985008, GU985009
10	TTC1	6A15	Tetratricopeptide repeat protein 1	408	136	330	F CAAGCACCACTGGAACGACGATAG<br>R GACGGAGAGGAACACAACAAAG	GU985067, GU985068, GU985069, GU985070, GU985071, GU985072, GU985073, GU985074, GU985075, GU985076, GU985077, GU985078, GU985079, GU985080, GU985081, GU985082, GU985083, GU985084, GU985085
11	STI1	HS718	Hsp70/Hsp90 organizing protein homolog	669	223	459	F GGGCCTTTTCACACGCTGCCTTA<br>R AAAGACGAGAAGGACGACGA	GU985048, GU985049, GU985050, GU985051, GU985052, GU985053, GU985054, GU985055, GU985056, GU985057, GU985058, GU985059, GU985060, GU985061, GU985062, GU985063, GU985064, GU985065, GU985066
12	CCT4	TCP1delta	T-complex protein 1, delta subunit	231	77	183	F CCTTCCAAGCAGGACAGCACCA<br>R TACACCCTCGCGGAGAAC	GU984839, GU984840, GU984841, GU984842, GU984843, GU984844, GU984845, GU984846, GU984847, GU984848, GU984849, GU984850, GU984851, GU984852, GU984853, GU984854, GU984855, GU984856, GU984857
13	CCT-epsilon	TCP1epsilon	T-complex protein 1, epsilon subunit	432	144	354	F AAAGCCCCTCGCGATGTTTATCGT<br>R GAGGCGAAACGCTCTCTC	GU984858, GU984859, GU984860, GU984861, GU984862, GU984863, GU984864, GU984865, GU984866, GU984867, GU984868, GU984869, GU984870, GU984871, GU984872, GU984873, GU984874, GU984875, GU984876
14	PXMP2/4_1	1E12	PXMP2/4 family protein 2	540	180	210	F AAAGAAGCCGCCGCAGTTGGTGTA<br>R GGTACACCGCTTTGCTAGAGT	GU985010, GU985011, GU985012, GU985013, GU985014, GU985015, GU985016, GU985017, GU985018, GU985019, GU985020, GU985021, GU985022, GU985023, GU985024, GU985025, GU985026, GU985027, GU985028
